# Functional Outcome After Decompressive Craniectomy in Patients with Dominant or Non-Dominant Malignant Middle Cerebral Infarcts

**DOI:** 10.7759/cureus.997

**Published:** 2017-01-26

**Authors:** Bilal Kamal Alam, Ahmed S Bukhari, Salman Assad, Pir Muhammad Siddique, Haider Ghazanfar, Muhammad Junaid Niaz, Maryam Kundi, Saima Shah, Maimoona Siddiqui

**Affiliations:** 1 Department of Internal Medicine, Fairview Hospital, Cleveland Clinic, USA; 2 Research Associate, Department of Neurology, Shifa International Hospital, Islamabad, Pakistan; 3 Department of Medicine, Shifa Tameer-e-Millat University, Islamabad, Pakistan; 4 Department of Internal Medicine, Shifa International Hospital, Islamabad, Pakistan; 5 Department of Neurology, Shifa International Hospital, Islamabad, Pakistan; 6 Department of Genito-urinary Oncology, Weill Medical College of Cornell University; 7 Department of Internal Medicine, Carthage Area Hospital, New York, USA; 8 Department of General Medicine, Hayatabad Medical Complex, Peshawar, Pakistan; 9 Consultant Neurologist, Department of Neurology, Shifa International Hospital, Islamabad, Pakistan

**Keywords:** decompressive craniectomy, functional outcome, ischemic stroke

## Abstract

Background: The use of decompressive craniectomy (DC) has been studied in the setting of different conditions, including traumatic brain injury, subarachnoid hemorrhage, and malignant middle cerebral artery (MCA) infarction. The rationale of this study is to determine the functional outcome after DC in patients with malignant MCA infarcts.

Methods: A longitudinal cohort study was performed based on patients diagnosed with malignant MCA territory infarction admitted to the Neurosurgery Department of a tertiary care hospital in Islamabad, Pakistan between July 2015 and November 2016. All patients had a clinical diagnosis of stroke according to the World Health Organization (WHO) stroke criteria.

Results: A total of 34 patients participated in this study, out of which 20/31 (64.5%) were males while 11/31 (35.5%) were females with a mean age of 51.61 ± 13.96 years. The mean time from diagnosis to surgery was 60.61 ± 49.83 hours. Out of 31 patients, 18 (58.1%) had a right middle cerebral artery infarct (RMCAI) and 13 (41.9%) had a left middle cerebral artery infarct (LCAI). Logistic regression was applied to assess the association between the type of MCA infarct with the National Institutes of Health Stroke Scale (NIHSS), modified Rankin Scale (mRS), modified Barthel Index (mBI) scores, and upper and lower limb motor power. However, the logistic regression model was not statistically significant χ^2^ (4) = 3.896, p = 0.866. There was a statistically significant mild improvement of neurological scores and upper and lower motor power over a course of six months, but the overall functional outcome was poor with mBI < 60 and mRS > 4 (p < 0.001) with total mortality of 8.7%.

Conclusion: Decompressive craniectomy is a life-saving surgery that appears to benefit patients with malignant MCA infarcts of either the dominant or non-dominant cerebral hemisphere. Decompressive craniectomy results in mild improvements in neurological scores but still poor functional outcome after six months.

## Introduction

Malignant middle cerebral artery (MCA) infarction accounts for approximately 10% of all patients who present with ischemic stroke with a mortality rate up to 80% [1]. Space-occupying edema in more than 50% to 75% of the middle cerebral artery region is known as malignant cerebral infarction [2]. Decompressive craniectomy (DC) is a life-saving surgery for patients with space-occupying hemispheric infarction due to limited medical treatments [3-5]. Cerebral edema in association with infarcted brain tissue causes displacement of brain tissue and leads to an increase in intracranial pressure (ICP). This secondary damage can be prevented by DC, which generates compensatory space to contain the swollen brain. The initial symptoms and signs of MCA occlusion are hemiparesis, hemiplegia, visual problems, and altered consciousness [6]. However, these patients worsen rapidly within the first 48 hours due to the presence of mass effect that can have serious consequences [7]. Intensive medical management with osmotic diuretics, sedation, hypothermia, hyperventilation, and mechanical ventilation have been unsuccessful so far, with an 80% reported mortality rate despite medical management [8-9].

Early critics documented the poor functional outcome of DC, particularly with dominant hemisphere involvement, resulting in a higher percentage of surviving patients who were dependent on caregivers, increasing their burden. In addition, the surgical complications and the cosmetic malformations were undesirable. Neurosurgeons may be hesitant to perform this procedure due to the lack of literature available on functional outcomes after DC. Recently, the pooled analysis of three small randomized trials (RCTs) showed that decompressive craniectomy can decrease mortality and morbidity associated with malignant infarction of the MCA [10]. Three renown RCTs, the French DECIMAL (the DEcompressive Craniectomy In MALignant MCA Infarction) trial, the Dutch HAMLET (Hemicraniectomy After Middle Cerebral Artery infarction with Life-threatening Edema Trial), and the German DESTINY (Decompressive Surgery for the Treatment of Malignant Infarction of the Middle Cerebral Artery) trial, demonstrated a major decline in mortality rates and improvement in functional outcome in patients managed with DC as compared to medical therapy [11-13]. Our study was conducted to compare the difference in functional outcomes in terms of mortality rate at 30 days and functional outcome at discharge, three months, and six months following DC for the treatment of malignant MCA infarction and medical treatment, as well as to study the association of factors influencing the outcome in patients treated with DC.

## Materials and methods

### Study design

A longitudinal cohort study of patients diagnosed with malignant MCA territory infarction admitted to the neurosurgery department of Shifa International Hospital, Islamabad, Pakistan between July 2015 and November 2016 was performed. Data were collected from patients' medical, surgical, and radiological imaging records. A total of 125 patients between the ages of 18 and 65 years were included in this study. All patients had a clinical diagnosis of stroke according to the World Health Organization (WHO) stroke criteria [14]. Malignant MCA territory infarction was defined as an infarct of at least two-thirds^ ^of the MCA region with space-occupying cerebral edema and mass effect on non-contrasted computed tomographic (CT) imaging of the brain. Pretreatment clinical evaluation was based on the Glasgow Coma Scale (GCS). Inclusion and exclusion criteria are shown in Table 1. Surgical treatment consisted of 10 x 12 cm diameter standardized DC with duraplasty. All patients were shifted in the neurosurgery intensive care unit (ICU) after surgery for an approximate duration of 24 - 48 hrs, and the decision regarding weaning of therapy was based on clinical as well as serial CT scan and magnetic resonance imaging (MRI) findings.

**Table 1:** Eligibility Criteria MCA: middle cerebral artery; mRS: modified Rankin Scale

Inclusion Criteria
Age between 10 - 60 years
National Institutes of Health Stroke Scales (NIHSS) > 15
Infarct > 50% of the MCA territory (or approximately > 145 ccs)
Decompressive craniectomy done within 12-48 hours of symptom onset
Written informed consent by the patient or legal representative
Decrease in level of consciousness (NIHSS ≥ 1 in 1a level of consciousness)
Glasgow Coma Scale scores (GCS) ≤ 8
Exclusion Criteria
Age < 10 and > 60 years
Life expectancy < 3 years
Both pupils fixed and dilated
Pre-existing significant disability (mRS > 2)
Significant contralateral infarct
Glasgow coma scale (GCS ≥ 9)
Significant contralateral infarction
Pregnancy
Contraindications of anesthesia

### Data collection

We collected data, such as age, sex, past medical history, distribution of the infarction, affected hemispheric dominance, National Institutes of Health Stroke Scale (NIHSS), modified Rankin Scale (mRS) and modified Barthel Index (mBI) scores. Informed consent was taken from patients before participation in the study. Approvals from the institutional review board and ethical committee of Shifa International Hospital, Islamabad, Pakistan were obtained for this cohort study (approval #486-336-2016).

### Outcome measurements

The outcome on follow-up was analyzed during outpatient department (OPD) visits in the stroke clinic and/or telephonically at three, six, and 12 months. Patients' outcomes were defined by rating the activities in their daily life with measurements established in stroke research by the mRS, NIHSS, and mBI. The mBI score of ≥ 60 and an mRS score of 3 or less were taken as favorable outcomes characterized by minimal-to-moderate disability, whereas an mBI less than 60 and an mRS of 4–6 define poor outcomes with moderately severe or very severe disability [15].

### Statistical analysis

Sample size calculation was done using power and sample size calculation software [16], with a level of significance (α) of 0.05 and a power of 0.80. Data entry and analysis were done using the Statistical Package for Social Sciences (SPSS) Software, version 22 (IBM Corp., NY, USA). Paired sample T-test at admission and at six months was used to determine significant differences in functional outcome scores and motor power of all limbs. Logistic regression and chi-square analysis were used to appreciate differences at admission, discharge, and at one, three, and six months between mRS, Barthel index, and motor power of the upper and lower limbs. A Spearman’s correlation was run to determine the relationship time to surgery with the duration of ICU stay and duration of hospital stay.

## Results

Data from a total of 34 patients was reviewed and analyzed. Out of these, 31 were selected while three were lost to follow-up. Twenty of the 31 patients (64.5%) were males while 11/31 (35.5%) were females. The mean age of all patients was 51.61 ± 13.96 years. The mean time to surgery was 60.61 ± 49.83 hours. Out of 31 patients, 18 (58.1%) had right middle cerebral artery infarcts (RMCAI) and 13 (41.9%) had left middle cerebral artery infarcts (LMCAI). The total hospital mortality rate was found to be 3/31 (8.7%). The mean duration of hospital stay was 15.5 ± 11.5 days. The mean number of days spent in intensive care unit (ICU) was 4.00 ± 2.58 (Table 2). A total of 23 patients were put on a mechanical ventilator at some point during their stay in the hospital. The mean number of hours on mechanical ventilator of these patients was calculated to be 58.48 ± 59.08.

**Table 2:** Patient Characteristics

Gender (N = 31)	
Male Females	20 (64.5%) 11 (35.5%)
Age (years)	
Mean ± standard deviation	51.61 ± 13.96 years
Hemisphere Infarct (N = 31)	
Right Left	18 13
Time from diagnosis to Surgery (Hours)	
Mean ± standard deviation	60.61 ± 49.83 hours
Duration of Hospital Stay	
Mean ± standard deviation	15.32 ± 11.36 days
Duration of ICU Stay	
Mean ± standard deviation	4.00 ± 2.58 days
Rate (%)	
Mortality rate Survival rate	8.7% 91.3%

The functional neurological scores of upper and lower limb motor power at admission, discharge, at one month, at three months, and at six months are shown in Table 3.

**Table 3:** Functional Neurological Scores of Upper and Lower Limb Motor Power at Admission, Discharge, at One Month, at Three Months, and at six months mRS: modified Rankin Scale; GCS: Glasgow coma scale; NIHSS: The National Institutes of Health Stroke Scale

Duration	mRS	GCS	Modified Barthel Index	NIHSS	Motor Power Upper Limb	Motor Power Lower Limb
At Admission	4.71±0.46	9.52±2.36	17.42±9.65	17.10±3.22	0.42±0.76	0.90±1.04
At Discharge	4.10±0.307	11.74±3.66	31.77±13.01	12.52±2.57	0.45±0.62	0.77±0.669
At One Month	4.06±0.25	12.06±3.40	42.58±13.22	11.90±2.33	0.74±0.68	1.19±0.79
At Three Months	4.03±0.32	12.26±3.17	49.19±13.73	11.13±2.62	1.19±0.79	1.42±0.81
At Six Months	4.03±0.31	12.35±3.09	55.65±13.46	10.45±2.23	2.03±1.02	2.65±1.08

**Figure 1:**
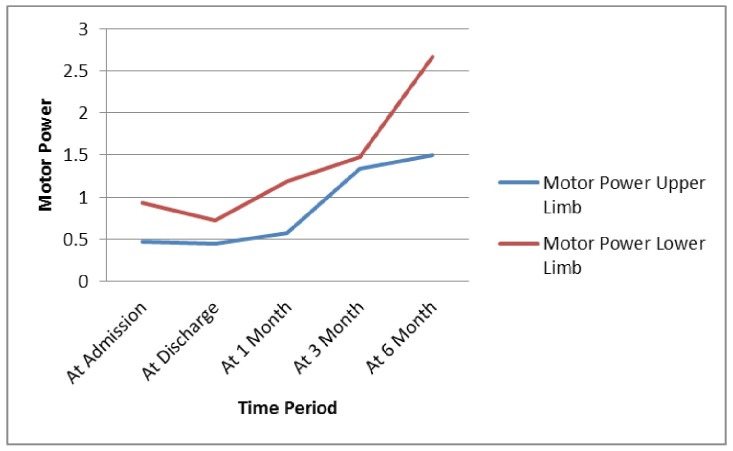
Changes of Upper and Lower Limb Motor Power with Passage of Time

At the time of discharge, 18/31 (51.8%) had dysarthria and 11/31 (35.5%) had aphasia. After one month, 16/31 (51.6%) had dysarthria and 10/31 (32.3%) had aphasia. After three months, 6/31 (19.4%) had dysarthria and 5/31 (16.1%) had aphasia. After six months follow-up, 4/31 (12.9%) had dysarthria and 2/31 (6.4%) had aphasia. Changes of upper and lower limb motor power with the passage of time are shown in Figure 1.

Logistic regression was applied to assess the association between the type of MCA infarct with mRS, GCS, NIHSS, modified Barthel Index, and upper and lower limb motor power. The logistic regression model was not statistically significant χ2 (4) = 3.896, p = 0.866. Pair-sample T-test was done to assess for any change in neurological score and upper and lower limb motor power at admission and six months after discharge. There was a statistically significant improvement of neurological scores and upper and lower motor power over a course of six months after treatment (p < 0.001) with poor functional outcomes (mRS > 4 and mBI > 60) (Table 4).

**Table 4:** Comparison of Different Neurological Functional Scores at Admission and After Six Months of Discharge *P-value derived from paired sample t-test mBI: modified Barthel Index; mRS: modified Rankin Scale; GCS: Glasgow coma scale; NIHSS: The National Institutes of Health Stroke Scale

Neurological Scores	Mean Difference ± SD	95% Confidence Interval of the Difference		t-value	P-value*
		Lower Limbs	Upper Limbs		
mRS at admission and at six months after discharge	0.68±0.54	.479	.876	6.97	< 0.001
mBI at admission and at six months after discharge	-38.23±11.66	-42.502	-33.950	-18.26	< 0.001
NIHSS at admission and at six months after discharge	6.65±3.49	5.365	7.924	10.61	< 0.001
GCS at admission and six months after discharge	-2.84±2.58	-3.786	-1.89	-6.12	< 0.001
Motor power of upper limb at admission and six months after discharge	-1.61±1.26	-2.074	-1.152	-7.15	< 0.001
Motor power of lower limb at admission and six months after discharge	-1.74±1.41	-2.260	-1.223	-6.86	< 0.001

A Spearman’s correlation was run to determine the relationship time to surgery with duration of ICU stay and duration of total hospital stay. There was a strong negative correlation between time from diagnosis to surgery and duration of ICU stay, which was statistically significant (rs = -0.429, p = 0.020). No statistically significant relationship was found between time to surgery and duration of hospital stay (rs = -0.230, p = 0.221). One-way analysis of variance (ANOVA) was applied to look for an association between the time from diagnosis to surgery and language impairment, and it showed no significant association (F = 0.613, p > 0.05). Of the three patients that expired, two were males and one was female. Among the expired patients, one patient had a right-sided MCA, whereas the other two had left-sided MCA cerebral infarcts. All three patients had an mRS at admission of 5 and a mean GCS of 7.67 ± 2.082.

## Discussion

The imperative role of DC in the survival of patients with malignant MCA infarction is widely accepted. Several randomized controlled trials have demonstrated a decrease in mortality of patients undergoing DC [11-13]. However, the issue more pertinent to current researchers is whether this life-saving surgery improves the quality of life and functional outcome of surviving patients. The results of our study demonstrate improvement in the neurological outcomes at one, three, and six months on mRS, mBI, GCS, and NIHSS scale, but the overall functional outcome of our patients was still graded as poor according to the mBI (less than 60) and mRS scale (> 4) at follow-up [14-15]. The major factors affecting functional outcome after the surgery include the timing of the surgery, preoperative condition of the patient, and the age of the patient [16]. The ideal time for undertaking the surgery should be within 48 hours of the onset of the stroke [17-18]. Also, severe neurological deficits at the time of admission and old age are considered to be bad prognostic factors for the long-term functional outcome after DC [19]. The mean time from diagnosis to surgery in our study was 60.61 hours, which may be the probable explanation for the relatively poor outcome of our patients on mBI and mRS at six months of follow-up. Despite the fact that the majority of our patients fall into the ideal age category for DC, severe neurological insufficiency at the time of admission and longer duration of mechanical ventilation may be responsible for the relatively poor functional outcome on various neurological scales [20].

One of the striking findings of our study is the considerable improvement in the motor power of both upper and lower limbs, but more so in the lower limbs. Carter, et al. concluded that the one-year outcome in the majority of their patients was noted to be at the level of near-independence, being able to walk, and having a Barthel score greater than 60 [21]. We expect nearly the same level of functional independence in our patients. In our study, statistically significant improvements in the neurological scores, along with upper and lower motor powers, were noted after six months of DC. The prominent finding of this study is a major improvement observed in dysarthria (from 51.8% patients at admission to 12.9% after six months) and aphasia (from 35.5% at admission to 6.4% patients after three months). However, we were unable to elaborate the dominant hemisphere and the type of aphasia in individual patients.

Our results comply with other studies, including a meta-analysis by Foerch, et al. According to that study, there was a considerable decrease in the mortality and improvement of functional outcome after DC as compared to the conservative management of malignant MCA infarct. However, it was still associated with an increased incidence of major disability (mRS score: 4-5) among the survivors [22]. Further evaluation about the exact type of functional disability among the survivors needs more attention. Our findings (mRS: 4-5) are in accordance with that of Suyama, et al. who determined the mRS score at three months in about half of their patients, out of whom only a small number of patients showed a favorable functional outcome [23]. This similarity is possibly due to delay in undertaking the surgery in a significant number of patients, i.e. after 48 hours of onset of stroke and low GCS scores preoperatively. In contrast to the mean age of 51.61 years in our study, the vast majority of their patients were elderly with the mean age of 67.0 years; however, age was not established as an independent factor for mortality in that study, justifying the use of surgery in elderly patients if further evidence of functional improvement is established [23].

### Limitations 

Neuropsychological evaluation of the patients could not be carried out on follow-up. Previous literature shows some depressive inclination in patients after undergoing the surgery [24]. The probable reason for improved clinical and functional outcomes after DC is due to the inclusion of patients under age of 60 years as literature already documents the poor outcome of DC in malignant MCA infarcts after 60 years of age [25]. Further research needs to be done on overall long-term neuropsychological assessment in patients after undergoing DC for malignant MCA infarcts.

## Conclusions

Decompressive craniectomy shows mild improvements in functional outcomes after six months of malignant MCA infarcts but with poor functional outcomes as a whole (mRS > 4 and mBI < 60). An early diagnosis is essential for DC, which has been shown to reduce mortality.
